# Therapeutic benefits of katathym visualization technique on elderly bio-psychological concerns

**DOI:** 10.6026/973206300212566

**Published:** 2025-08-31

**Authors:** Packiyalakshmi Muthuchamy, Vasudevan Venkatesh Mathankumar, Shankar Shanmugam Rajendran, Marudan Anbalagan, Balasubramaniyan Srividhya, Elilarasi Mani, Gomathi Sivaprakash

**Affiliations:** 1 Department of Psychiatric Nursing, College of Nursing, Madras Medical College, The Tamil Nadu Dr. MGR Medical University, Chennai, Tamil Nadu, India; 2 Department of Psychiatry, Institute of Mental Health, Chennai, Tamil Nadu, India; 3 Department of Child Health Nursing, College of Nursing, Madras Medical College, The Tamil Nadu Dr. MGR Medical University, Chennai, Tamil Nadu, India; 4 Department of Child Health Nursing, College of Nursing, Madras Medical College, The Tamil Nadu Dr. MGR Medical University, Chennai, Tamil Nadu, India; 5 Department of Psychiatric Nursing, College of Nursing, Madras Medical College, The Tamil Nadu Dr. MGR Medical University, Chennai, Tamil Nadu, India

**Keywords:** Katathym visualization technique, bio-psychological concerns, elderly, old age homes

## Abstract

The effectiveness of Katathym Visualization Technique (KVT) in improving sleep quality and reducing psychological distress among elderly individuals in old age homes is of interest. A quasi-experimental design was conducted in Chennai with 60 participants, who were assigned to either the experimental (KVT) or control group. After four weeks of daily KVT sessions, 76.67% of the experimental group reported an improvement in sleep quality and 80% reported a reduction in psychological distress. KVT proved to be effective, suggesting its potential integration into elderly care. Future research should explore long-term effects and compare KVT with other interventions.

## Background:

With the aging of the global population, mental well-being has become a vital aspect of elderly health [1]. Elderly individuals, particularly those in institutional settings such as old age homes, are highly susceptible to emotional distress, anxiety, loneliness and disturbed sleep due to declining health and lack of social support. These issues significantly diminish quality of life and emotional balance [2]. In India, the elderly population is expected to exceed 300 million by 2050, with studies showing high rates of mental health issues among residents in care homes [3]. Tamil Nadu mirrors this trend, with nearly 18% of its population projected to be over 60 by 2031. Institutionalized elders in Chennai often lack access to structured psychological care, highlighting the urgent need for effective, non-pharmacological interventions [4]. The Katathym Visualization Technique (KVT), also known as guided affective imagery, is rooted in depth psychology and involves symbolic visualization to help individuals access unconscious emotions, reduce internal conflict, and promote relaxation [5]. KVT has been recognized internationally for its therapeutic potential; however, its application in Indian geriatric care remains limited [6]. Therefore, it is of interest to describe the potential of the Katathym Visualization Technique (KVT) as an effective non-pharmacological intervention for improving the mental well-being and sleep quality of elderly individuals in institutional care settings.

## Materials and Methods:

Quasi-experimental designs to evaluate the impact of the Katathym Visualization Technique (KVT) on sleep patterns and psychological distress in elderly residents of old age homes. The research was conducted in two selected geriatric institutions in Chennai: Birds Nest Old Age Home (Avadi) and Sundari Ammal Old Age Home (Ayapakkam), with ethical clearance from the Institutional Ethics Committee of Madras Medical College (Ref No: IEC/MMC/52112024). A total of sixty elderly participants aged 60 and above were chosen using purposive sampling. They were divided into two groups: the experimental group received planned KVT sessions, while the control group received standard routine care. KVT was administered over four weeks, with daily sessions of 30-40 minutes involving guided symbolic imagery and emotional exploration to promote inner calmness, emotional insight and relaxation. Data were collected before and after the intervention using validated instruments: the Geriatric Sleep Questionnaire for sleep evaluation and the Kessler Psychological Distress Scale (K10) for assessing emotional symptoms. Demographic and health-related information, including age, sex, comorbidities, physical activity and duration of institutional stay, was also recorded. The gathered data were analysed using IBM SPSS. Chi-square testing was used to assess categorical data, while paired and independent t-tests were employed to examine differences in numerical data between the groups. A P-value of ≤ 0.05 was used to determine statistical significance.

## Results:

The study findings revealed based on demographic variables, most elderly were aged 71-75 years (53.33% experimental, 46.67% control), male (66.67%, 53.33%), educated up to 10th standard (43.34%, 40%), with children as income source (40%, 53.34%), and voluntary admission (63.33%, 70%).In the pre-test, the experimental group had 10% good sleep and 60% severe distress, while the control group had 13.33% good sleep and 70% severe distress. In the post-test, the experimental group improved to 70% good sleep and 60% mild distress, whereas the control group had 56.67% poor sleep and 60% severe distress (Table 1 - see PDF, Figure 1 - see PDF). Table 1 (see PDF) and Figure 1 (see PDF) show that in the experimental group, the mean psychological distress score decreased from 33.5 ± 6.1 in the pre-test to 20.97 ± 3.5 in the post-test, with a mean difference of 12.53 (t = 9.76, p = 0.001), indicating a highly significant reduction. In the control group, the reduction from 34.67 ± 4.9 to 32.97 ± 4 had a mean difference of 1.7 (t = 1.86, p = 0.07), which was not significant. Table 2 (see PDF) and Figure 2 (see PDF) show that in the experimental group, the sleep score improved from 18.83 ± 4.3 in the pre-test to 11.37 ± 2 in the post-test, with a mean difference of 7.46 (t = 9.61, p = 0.001), showing a very highly significant improvement. The control group's change from 19.07 ± 4 to 17.83 ± 3.5 had a mean difference of 1.24 (t = 1.74, p = 0.09), which was not significant (Table 2 - see PDF, Figure 2 - see PDF). Associations were found with variables such as age, type of admission, presence of illness, and physical activity.

## Discussion:

The present study found that the Katathym Visualization Technique (KVT) significantly improved sleep quality and reduced psychological distress among elderly residents in old age homes. These findings align with earlier studies, including those by Amini *et al.* [7], which documented enhanced emotional stability and decreased anxiety through the use of guided imagery. A negative correlation observed between sleep quality and psychological distress further underscores the emotional benefits associated with better rest. The results are also supported by Wang *et al.* (2021) [8], who emphasized the role of KVT in promoting emotional processing through symbolic visualization. Similarly, Geiger *et al.* (2025) [9] demonstrated that mindfulness-based practices significantly reduce stress and depression among elderly individuals in institutional settings. Collectively, these studies underscore the importance of incorporating non-pharmacological interventions, such as KVT, into standard geriatric care to enhance emotional well-being.

## Conclusion:

Nurses play an essential role in implementing and promoting Katathym Visualization Technique for elderly individuals in long-term care settings. By incorporating guided imagery practices, nurses can help reduce psychological distress and improve sleep quality, thereby enhancing the overall well-being of elderly residents. Their involvement in patient education and continued emotional support strengthens the effectiveness of such interventions, fostering a holistic approach to geriatric mental health care.
